# Unlocking heart anti-aging potential: the SIRT2-STAT3-CDKN2B pathway as a bridge between fiction and reality

**DOI:** 10.1093/lifemedi/lnae020

**Published:** 2024-05-13

**Authors:** Chunyu Chen, Jingxuan Liu, Wei Yu

**Affiliations:** State Key Laboratory of Genetic Engineering, School of Life Sciences, Zhongshan Hospital, Fudan University, Shanghai 200438, China; State Key Laboratory of Genetic Engineering, School of Life Sciences, Zhongshan Hospital, Fudan University, Shanghai 200438, China; State Key Laboratory of Genetic Engineering, School of Life Sciences, Zhongshan Hospital, Fudan University, Shanghai 200438, China

As the global aging population continues to expand, the impact of age-related cardiovascular diseases (CVDs) on health and longevity has garnered increasing concern. According to data from the World Health Organization, CVDs accounted for an estimated 17.9 million global deaths in 2019, constituting 32% of all worldwide mortality. Consequently, there is a pressing need to delve into the molecular underpinnings of cardiac aging to pave the way for future advancements in preventive and therapeutic approaches.

Sirtuins, known as silent information regulator 2 proteins, constitute a family of histone deacetylases (HDACs) responsible for catalyzing the deacetylation of both histone and non-histone lysine residues. Beyond their deacetylase activity, certain sirtuins also exhibit adenosine diphosphate (ADP)-ribozyme, demalonylase, desuccinylase, or glutarylase properties [1]. Among the Sirtuin family, SIRT2, situated in the cytosol, remains relatively less explored. It was found expression in a broad spectrum of tissues, with a prominent presence in metabolism-related tissues, such as the heart, brain, and adipose tissue. SIRT2 plays a pivotal role in various metabolic processes. Over the past years, researchers have unveiled its significant contributions to cardiovascular diseases. For instance, SIRT2 functions as a cardioprotective deacetylase, guarding against aging-dependent and Ang II-induced pathological hypertrophy by sustaining cardiac LKB1-AMPK signaling. Comparatively, SIRT2-knockout mice, in contrast to their wild-type counterparts, displayed exacerbated cardiac hypertrophy, fibrosis, and compromised cardiac function following angiotensin II infusion. These findings underscore the potential of SIRT2 as a therapeutic target for addressing pathological cardiac hypertrophy [2]. Furthermore, SIRT2 has been implicated in age-dependent vascular aging. As individuals age, both SIRT2 protein levels and its activity tend to diminish, and the deficiency of SIRT2 exacerbates vascular dysfunction and remodeling in elderly mice. Researchers have elucidated that SIRT2 contributes to the regulation of vascular aging, in part, by inhibiting the aging-associated protein, p66^Shc^, and its downstream mitochondrial reactive oxygen species (mROS). This discovery unveils an unforeseen role for the deacetylase SIRT2 as a guardian against vascular aging and underscores the significance of the cytoplasm-mitochondria axis involving SIRT2-p66Shc-mROS in the context of age-induced vascular remodeling [3]. SIRT2 also plays a vital role in peripheral neurological diseases. Wild-type GARS binds to SIRT2, inhibiting its deacetylation activity and leading to increased acetylated α-tubulin levels, a major SIRT2 substrate. The catalytic domain of GARS, frequently associated with Charcot-Marie-Tooth type 2D (CMT2D) mutations, interacts closely with SIRT2. However, CMT2D mutations in GARS fail to inhibit SIRT2 deacetylation, resulting in decreased acetylated α-tubulin levels. Reducing SIRT2 genetically in a *Drosophila* model has been effective in mitigating GARS-induced axonal CMT neuropathy and extending lifespan, offering a potential treatment avenue for hereditary peripheral nerve diseases [4]. Nonetheless, all of the aforementioned studies were conducted in non-primate models. This raises the question of the molecular mechanisms underlying cardiac pathophysiological changes induced by aging in primates.

**Figure 1.**
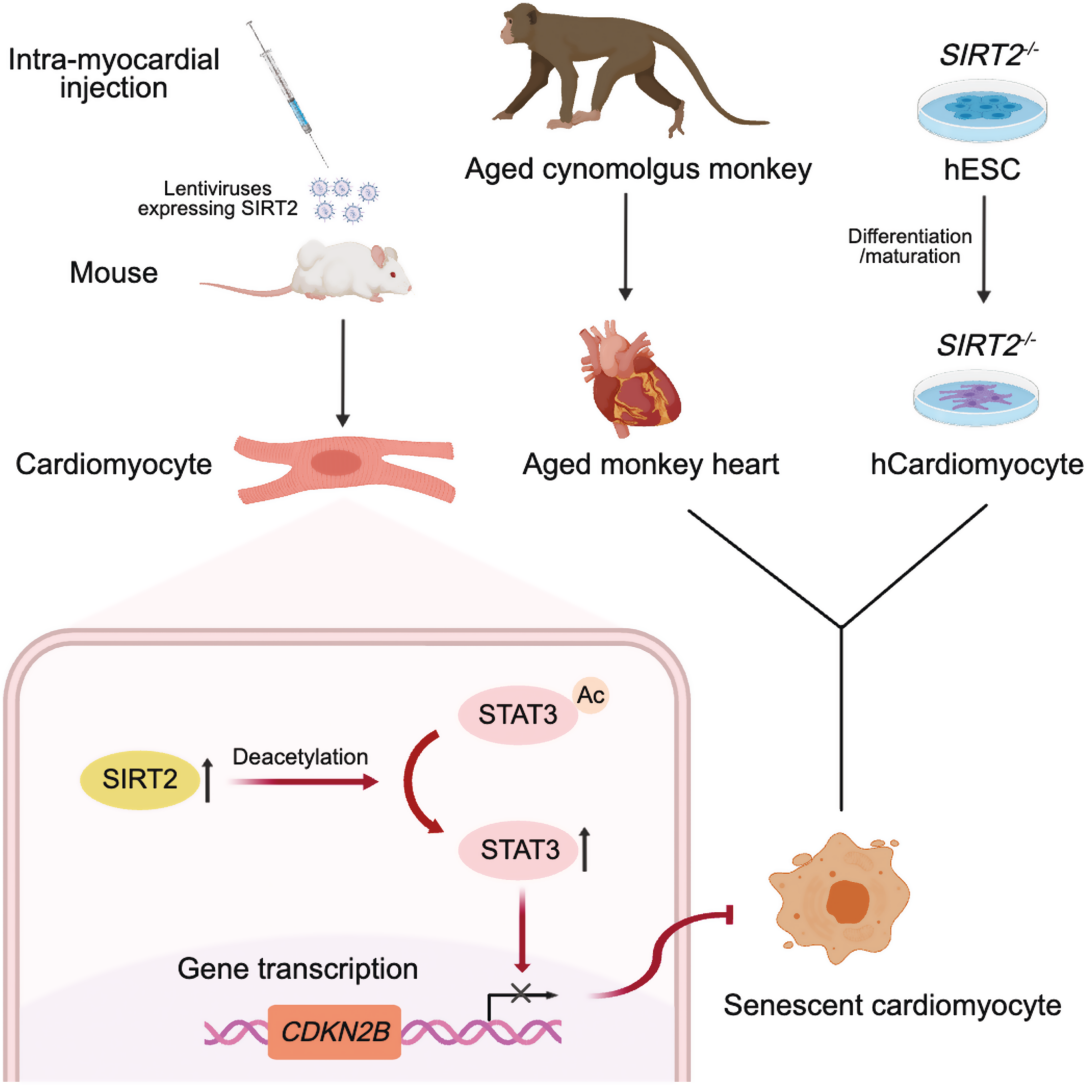
The role of SIRT2 in cardiac aging. (Contributed by Jingxuan Liu)

In a groundbreaking study led by Guang-Hui Liu’s team [5], a milestone was reached as they conducted a comprehensive analysis of the transcriptome and proteome of aging hearts in cynomolgus monkeys. For the first time, this research unveiled the pivotal biological pathway driving cardiac aging in primates, namely the SIRT2-STAT3-CDKN2B pathway. This discovery fills a critical gap in our understanding of age-related cardiac physiological changes in primates. Moreover, the study demonstrated the potential of reversing age-related heart dysfunction through SIRT2 gene therapy (Fig. 1).

In this study, researchers employed a naturally aging model using cynomolgus monkeys. They systematically investigated aging-related phenotypes, including cardiac hypertrophy, sarcomeric abnormalities, and inflammation. Their approach encompassed high-throughput proteome and transcriptome sequencing, gene editing techniques, experimentation with human pluripotent stem cell-derived cardiomyocytes, gene expression modulation, and the application of viral vectors for gene therapy. Their findings pointed to a crucial factor in primate cardiomyocyte aging: the down-regulation of SIRT2 protein expression.

Initially, the researchers conducted a comprehensive analysis to identify aging-associated proteins. Remarkably, they found that the deacetylase SIRT2 was the sole protein exhibiting down-regulation during aging, and it was linked to both various cardiovascular diseases and epigenetic regulation. This discovery emerged from a joint analysis that encompassed differential senescence proteins discovered in cynomolgus monkey hearts and distinct sets of protein-coding genes. These gene sets included those related to senescence-related cardiovascular diseases, Aging Atlas senescence-related genes, and genes associated with epigenetic regulation. Furthermore, the researchers made a groundbreaking advancement by generating SIRT2-deficient human cardiomyocytes using CRISPR-Cas9-mediated gene editing, alongside human pluripotent stem cell-derived cardiomyocytes. This pioneering approach revealed that SIRT2-deficient human cardiomyocytes displayed a range of cardiac senescence-related characteristics, such as accelerated senescence and abnormal hypertrophy. Importantly, their gene expression profiles closely mirrored those observed in the aging hearts of cynomolgus monkeys.

Next, the researchers delved deeper into the mechanisms underlying their findings through a combination of advanced techniques. They conducted a comprehensive analysis that included transcription factor network analysis, immunoprecipitation coupled with protein profiling, protein-targeted mutagenesis, chromatin immunoprecipitation, real-time quantitative polymerase chain reaction, and *in situ* electron microscopy. Their investigations unveiled a significant interaction between the SIRT2 protein and the transcription factor STAT3. This interaction prompted the deacetylation of STAT3 at K685, subsequently inhibiting the transcription and expression of the cell cycle-arresting gene *CDKN2B*. The absence of SIRT2 led to an increase in the acetylation level of STAT3 in human cardiomyocytes, resulting in the upregulation of the *CDKN2B* gene and, consequently, cardiomyocyte senescence and hypertrophy. In contrast, both the overexpression of SIRT2 and the knockdown of CDKN2B exhibited the ability to delay the senescence of human cardiomyocytes.

Having established that SIRT2 plays a pivotal role in regulating human myocardial senescence, the researchers hypothesized that gene therapies centered around SIRT2 could potentially delay cardiac senescence. To investigate this hypothesis, they conducted an experiment where they introduced lentiviruses carrying SIRT2 proteins into the myocardium of 24-month-old mice at multiple sites. Remarkably, after just 2 weeks, they observed substantial improvements in key cardiac parameters, including ejection fraction, fractional shortening, and the extent of cardiac hypertrophy in the aged mice. These findings suggest that SIRT2 holds significant promise as a critical target for *in vivo* interventions aimed at combating myocardial aging.

While this research has yielded exciting breakthrough results, there are avenues for further exploration and refinement: (i) Species differences: Future research should consider other primate models or conduct clinical studies in humans to better understand human heart aging. (ii) Mechanism analysis: Further study is needed to delve into the molecular interactions and regulation of the SIRT2-STAT3-CDKN2B pathway. (iii) Clinical challenges: Challenges in translating findings to clinical treatments require validation through clinical trials for effectiveness and safety.

In conclusion, this study identifies the SIRT2-STAT3-CDKN2B pathway as a key regulator of senescence in primate cardiomyocytes. This discovery advances our understanding of cardiac aging and offers potential therapeutic targets. The down-regulation of SIRT2 may serve as a biomarker of cardiac senescence, which may help to accurately assess the functional status of the heart and predict the risk of cardiac senescence-associated diseases and may lead to the development of individualized interventions to prevent and control diseases. Future research may lead to interventions and treatments, such as SIRT2-enhanced gene therapies or SIRT2-specific agonists, with the potential to extend a healthy lifespan and address cardiovascular diseases.
